# Impact of Raw, Roasted and Dehulled Chickpea Flours on Technological and Nutritional Characteristics of Gluten-Free Bread

**DOI:** 10.3390/foods11020199

**Published:** 2022-01-12

**Authors:** Gokcen Kahraman, Sebnem Harsa, Maria Cristina Casiraghi, Mara Lucisano, Carola Cappa

**Affiliations:** 1 Department of Food Engineering, Faculty of Engineering, Izmir Institute of Technology, Izmir 35430, Turkey; gokcenkomen@gmail.com; 2 Department of Food, Environmental and Nutritional Sciences (DeFENS), Università degli Studi di Milano, Via G. Celoria 2, 20133 Milan, Italy; maria.casiraghi@unimi.it (M.C.C.); mara.lucisano@unimi.it (M.L.); carola.cappa@unimi.it (C.C.)

**Keywords:** gluten-free bread, chickpea, crumb texture, staling, nutritional properties

## Abstract

The main objective of this study was to develop a healthy rice-based gluten-free bread by using raw, roasted, or dehulled chickpea flours. All breads containing chickpea flours showed a darker crust and were characterized by an alveolar (porosity 41.5–51.4%) and soft crumb (hardness 5.5-14.1 N). Roasted chickpea flour bread exhibited the highest specific volume, the softest crumb, and the slowest staling rate. Enriching rice-based breads with the chickpea flours resulted in increased protein (from 9.72 to 12.03–13.21 g/100 g dm), ash (from 2.01 to 2.45–2.78 g/100 g dm), fat (from 1.61 to 4.58–5.86 g/100 g), and total phenolic contents (from 49.36 up to 80.52 mg GAE/100 g dm), and in reduced (~10–14% and 13.7–17%, respectively) available starch levels and rapidly digestible starch compared to rice bread. Breads with roasted chickpea flour also showed the highest in vitro protein digestibility. The results of this study indicated that the enrichment of rice-based gluten-free breads with chickpea flours improved the technological and nutritional quality of the breads differently according to the processed chickpea flour used, also allowing recovery of a waste product.

## 1. Introduction

The market for gluten-free (GF) products is continuously expanding due to the increasing number of people who have dietary restrictions caused by celiac disease and non-celiac gluten sensitivity. Nutrient deficiencies are a problem in the GF diet, arising from the lack of protein, dietary fiber, vitamins, and minerals [1,2,3]. This might be attributed to the large use of refined flours and starches, especially in GF baked goods and pasta products [4]. Since bread can be considered a staple food for consumers, its fortification could considerably affect the nutrient intake of people suffering from gluten-related disorders. To this end, fortifying bread formulations with legumes could be a promising way to enhance the nutritional value of GF bread due to their high protein, mineral, and dietary fiber content [5].

According to legume production statistics for 2018, soybeans, beans, and chickpeas are the most cultivated legumes in the world [6]. India, Australia, Turkey, and the Russian Federation are the top four chickpea producers [6]. Chickpea flour is considered a good nutrient source, containing 22.39% protein, 57.82% carbohydrate, 10.80% total dietary fiber, and 6.69% fat (rich in polyunsaturated fatty acids), in addition to B complex vitamins and minerals according to the USDA nutrient database [7].

Chickpeas can be consumed in many different ways, boiled, roasted, pressure-cooked, or used as an ingredient in many food formulations after milling. The heat processing of chickpeas may cause some alterations in the structure, functional properties, and macronutrient bioavailability [8,9,10]. Additionally, as a result of roasting and milling, the hulls of the chickpea might be partially and/or completely lost. The removal of the hulls causes some reductions in the levels of mineral and dietary fiber. However, enhanced bioavailability of other nutrients can be observed due to the removal of phytic acid and tannins, which are the anti-nutritional factors mainly present in the hulls [11]. In our earlier publication [12], the positive effect of the addition of roasted, dehulled, and raw chickpea flour to GF dough was demonstrated in terms of enhanced viscoelastic properties and slower retrogradation tendency of the dough. Furthermore, the advantages of adding chickpea flour [13,14,15,16,17] and roasted chickpea flour [18] in breadmaking have been discussed in literature for gluten-containing bread.

To date, studies related to the development of yeast-leavened GF bread containing chickpea flour are limited [19,20,21], and few efforts on the use of untreated and treated (e.g., thermally treated) chickpea flours have been discussed [22,23].

This study investigates the effect of the addition of flours obtained from differently processed chickpea to a rice-based bread in terms of technological and nutritional quality. Untreated (i.e., raw), thermally treated (i.e., roasted), and refined (i.e., dehulled) chickpeas were used. The thermally treated chickpea flour used in this research represents a waste product of leblebi, a chickpea snack widely consumed in Turkey and nearby countries, manufacturing [16,17].

## 2. Materials and Methods

### 2.1. Materials

Rice flour (RF; Remyflo R7-200) and dehulled chickpea flour (DCF; Homecraft Pulse 4101) were provided by Beneo-Remy NV (Leuven-Wijgmaal, Leuven, Belgium) and Ingredion GmbH (Hamburg, Germany), respectively. Raw chickpeas (Yayla, Ankara, Turkey) and leblebi, or roasted chickpeas, (Tuğba, Aydın, Turkey) (same chickpea variety as DCF) were obtained from a local Turkish market and milled by a laboratory mill (Yüksel Kaya Makina, Ankara, Turkey) to obtain chickpea flour (CF) and roasted chickpea flour (RCF), with >95% of particles below 500 µm (measured by an analytical sieve shaker, Octagon Digital, Endecotts Ltd., London, UK), as reported in Kahraman et al. [12]. The roasted chickpeas were obtained after three tempering (preheating and resting) steps, followed by moistening, resting, and roasting steps, as described in [24].

A structuring and a thickening agent commonly used in GF bread production, hydroxypropylmethylcellulose (HPMC, Benecel F4M, Ashland, Covington, KY, USA), was used in the bread formulations. Instant yeast (Pakmaya, Istanbul, Turkey), sugar (Bal Küpü, Aksaray, Turkey), salt (Billur, İzmir, Turkey), and sunflower oil (Yudum, Balıkesir, Turkey) were the other ingredients used in the GF bread formulations.

### 2.2. Methods

#### 2.2.1. Flour Characterization

Protein, fat, and ash contents of RF and chickpea flours have already been reported in our previous study [12], while total starch was determined using the “Total Starch Assay Kit” (Megazyme International Ireland Ltd., Bray Business Park, Bray, Co. Wicklow, Ireland). All determinations were performed in triplicate. Since the dietary fiber assay is time-consuming and commercial flours were used, fiber contents were extrapolated directly from the sample data sheet in order to complete the proximate composition of RF and chickpea flours.

Furthermore, the flour phenolic compounds were evaluated as reported in Section 2.2.4.

#### 2.2.2. Bread Preparation

Ten bread loaves for four different GF bread formulations were prepared. For the GF reference bread (RF bread), the formulation contained only commercial rice flour. For the supplemented chickpea breads, 25% of the rice flour was replaced with RCF, CF, or DCF. In addition, the bread dough formulation included HPMC (1.7 g/100 g), sugar (2.0 g/100 g), salt (1.5 g/100 g), instant yeast (2.5 g/100 g), sunflower oil (5.3 g/100 g), and water (amounts required to reach 125 ± 5 Brabender units (BU) as reported by Kahraman et al. [12]). All these percentages were based on flour (RF or RF combined with differently processed chickpea flour) weight.

Dry ingredients were blended in a Hobart N-50 mixer (Hobart Corporation, Troy, OH, USA) equipped with a flat beater for 1 min at 281 rpm. Then, water and oil were added and mixed for 2 min at 281 rpm and 3 min at 580 rpm. The obtained dough (150 g) was placed in baking pans and proofed in a proofing cabinet (HC0020, Haereus Vötsch, Frommern, Germany) at 32 °C and 85% relative humidity (RH) for 30 min. The proofed loaves were placed in a preheated oven (Lotus P4, Treviso, Italy) and baked for 45 min at 225 °C (top) and 205 °C (bottom). The loaves were removed from the pans and cooled at room temperature for 2 h. The bread samples were stored in polyethylene bags at 20 °C and 60% RH for 23, 47, 71, and 95 h after baking.

#### 2.2.3. Technological Properties of GF Bread

Fresh bread was characterized in terms of bake loss (%), moisture (g/100 g; gravimetrically by drying the sample at 105 °C to constant weight), water activity (CX-2, AquaLab, Pullman, WA, USA), volume (cm^3^) determined by the rapeseed displacement method, and specific volume (cm^3^/g) calculated as the ratio between bread volume and weight. The height (cm) of each loaf was measured using a caliper. Moisture content and water activity were also analyzed at 23, 47, 71, and 95 h of storage.

Crust and crumb color parameters were measured by a colorimeter (Chroma Meter II Reflectance, Minolta, Osaka, Japan) in CIE-Lab color space where L* (Lightness ranged from 0 = black to 100 = white), a* (+a, red; −a, green), and b* (+b, yellow; −b, blue). The illumination system consisted of a high-power pulsed xenon arc lamp, using C illuminant. Before measurements, the colorimeter was calibrated using the standard white reflector plate (Y = 87.7; x = 0.308; y = 0.315). For each bread formulation, the color of 10 loaves was evaluated by applying the head of the colorimeter (8 mm in diameter) directly to the sample surface and taking three measurements per loaf (*n* = 30) for both crust and crumb.

Crumb microstructure was analyzed with environmental scanning electron microscopy (ESEM; Quanta 250 FEG, FEI, Hillsboro, OR, USA). Samples were cut and attached to double-sided carbon tapes without any pretreatment. Images were captured at a voltage of 10 kV and a pressure of 400 Pa.

Crumb porosity was evaluated according to Cappa et al. [25]. Two slices for each bread formulation were scanned at 600 dpi using a flatbed scanner (HP SCANJET 8300, Hewlett-Packard, CA, USA), selecting a crop area of 80% of the total slice area. Based on the crumb pore size (0.1≤ x <0.2 mm^2^, 0.2 ≤ x < 0.5 mm^2^, 0.5 ≤ x < 1 mm^2^, 1 ≤ x < 5 mm^2^, 5 ≤ x < 10 mm^2^, and x ≥ 10 mm^2^), the pores were classified into 6 groups. For each group, the number and area of the crumb pores were given as the percentage of the total number and crop area, respectively. The crumb porosity (%; expressed as the total alveolate area of the crop divided by the total surface) was determined.

The crumb texture of the bread was evaluated by texture profile analysis (TPA) with TA-HDplus (Stable Micro Systems, Surrey, UK) after 2 (fresh bread), 23, 47, 71, and 95 h from baking. The texture analyzer was equipped with a 50 kg load cell and a 25 mm cylinder probe. Bread slices of 25 mm thick were compressed in the center to 40% of their original height with a test speed of 1 mm/s. Hardness, cohesiveness, and springiness were calculated using software (Exponent software 6.1.9, Stable Micro Systems, Surrey, UK). Hardness (N) was the maximum peak force obtained in the first compression (Supplemental Figure S1). Springiness was calculated as the ratio of time for second compression (time 4–5) to first compression (time 1–2). Cohesiveness is the property regarding the disintegration of the tested food under mechanical action, and it is defined as the ratio of the positive force area during the second compression (area 4–6) to that during the first compression (area 1–3). At least six slices for each formulation were analyzed. The rate of staling ([Hardness_95h_ − Hardness_2h_]/Hardness_2h_) was also calculated [26].

#### 2.2.4. Nutritional Properties of GF Bread

The total nitrogen content of bread samples was determined according to the Official Standard Method AOAC 920.87 [27] by using a block digestion system (Kjeldatherm, Gerhardt, Königswinter, Germany) and a distillation system (Vapodest 50s, Gerhardt, Königswinter, Germany). To estimate the protein contents of rice and chickpea-containing breads, conversion factors of 5.95 and 6.25 were used, respectively. Fat content was measured by an automatic extraction system (Soxtherm, Gerhardt, Königswinter, Germany) on a 4 g sample with hexane. The ash content was analyzed according to AACC [28] using a muffle furnace (Protherm, Ankara, Turkey).

The phenolic compounds were extracted from flour and bread according to Alves et al. [29] with some modifications. Samples (1 g) were mixed with acetone (10 mL, 70%, *v/v*) (24201, Riedel-de Haën, Seelze, Germany), shaken (Reax 2, Heidolph, Schwabach, Germany) for 1 h at 400 rpm in the dark, and centrifuged (Universal 320R, Hettich, Germany) at 2860× *g*, at 20 °C for 5 min, and then the supernatant was transferred to another tube; this procedure was repeated 3 times, then all the supernatants were combined. Extractions were performed in duplicate. The total phenolic content (TPC) of the acetone/water extracts was determined according to Singleton et al. [30] and Sakač et al. [31] with some modifications. The extract (0.5 mL) was mixed with distilled water (7.5 mL) and Folin-Ciocalteu’s reagent (0.5 mL) (109001, Merck, Darmstadt, Germany). After 5 min, 1.5 mL of sodium carbonate (13418, Sigma-Aldrich, St. Louis, MO, USA) solution (20%, *w/v*) was added. The tubes were vortexed, put in a shaker (KS 130 Basic, IKA, Staufen, Germany) in a dark place, and shaken at 480 rpm for 120 min at room temperature. The absorbance values were recorded at 760 nm with a spectrophotometer (UV-1601, Shimadzu, Tokyo, Japan). The standard curve was prepared by using gallic acid (G7384, Sigma, St. Louis, MO, USA), and the results were expressed as gallic acid equivalent (GAE) (mg GAE/100 g sample, dry weight).

The slowly digestible starch (SDS) and rapidly digestible starch (RDS) of bread samples were assessed according to the method described by Englyst et al. [32]. RDS and SDS fractions are likely to become available for rapid or slow absorption from the small intestine, thus modulating glycemic response. Fresh bread samples were minced by using a manual meat mincer (particle size less than 0.9 cm), and aliquots containing an estimated amount (500–600 mg) of carbohydrates were treated with pepsin from porcine gastric mucosa (≥250 units/mg solid, P7000, Sigma, St. Louis, MO, USA). Then the material was incubated with a mixture of pancreatin from porcine pancreas (8 × USP, P7545, Sigma, St. Louis, MO, USA), amyloglucosidase from *Aspergillus niger* (A7095, Sigma, St. Louis, MO, USA) and invertase (I4504, Sigma, St. Louis, MO, USA) under controlled conditions of pH, temperature (37 °C), viscosity (by adding guar gum) and mechanical mixing (160 rpm) [32]. Rapidly (RDS) and slowly (SDS) digestible starch fractions were calculated from the glucose released at 20 min and between 20 and 120 min of incubation with the mixture of hydrolytic enzymes, respectively. Glucose was detected by HPLC Anion Exchange Chromatography with Pulsed Amperometric Detection (HPAEC-PAD) as reported by Englyst et al. [32]. The glucose contents were measured by High-Performance Liquid Chromatography (HPLC) (Series 200, Perkin Elmer, Norwalk, CT, USA) equipped with 4 × 250 mm CarboPac™ PA1 column (Dionex, Sunnyvale, CA, USA) and PAD detector (ED50, Dionex, Sunnyvale, CA, USA). The sample (20 μL) was injected into the column at room temperature using NaOH (160 mM) as the mobile phase at a flow rate of 1 mL/min. RDS and SDS fractions were expressed as the percentage of available starch (AVST = RDS + SDS).

The in vitro protein digestibility (IVPD) of bread samples was determined according to Hsu et al. [33] with some modifications. Oven-dried bread samples were ground with a mortar and sieved (≤1 mm) before the analysis. Samples were mixed with water to obtain 6.25 mg crude protein/mL and rehydrated at 4 °C for 1 h. The pH of the sample mix was adjusted to 8.0, and the temperature was set as 37 ± 0.3 °C. *Nα-p*-Tosyl-l-arginine methyl ester hydrochloride (TAME) (T4626, Sigma-Aldrich, St. Louis, MO, USA) was used as substrate. The trypsin activity of pancreatin (P7545, Sigma, St. Louis, MO, USA) was measured as 7.53 TAME units/mg according to the method described by Minekus et al. [34]. A proper amount of pancreatin (P7545, Sigma, St. Louis, MO, USA) was dissolved in 1 mM HCl in order to obtain 13,766 BAEE units/mg protein (1 TAME unit = 57.5 BAEE (*N*-benzoyl-l-arginine ethyl ester) units), and pH was adjusted to 8.0. The enzyme solution (5 mL) was added to the sample solution (50 mL), and the pH drop was recorded for 10 min. The pH of the solution immediately after 10 min of digestion with enzyme solution (x) was used to calculate the percentage of in vitro protein digestibility (Y) by using the equation: Y = 210.46 − 18.10x [33].

#### 2.2.5. Statistical Analysis

The results were given as mean ± SD. If not differently specified, measurements were done in duplicate on two bread loaves (n = 4). Statistical evaluation of the data was performed with MINITAB 16 (Minitab Inc., State College, PA, USA). Data were tested by analysis of variance (ANOVA) at *p* < 0.05, and means were compared by Tukey’s test at a 95% confidence interval.

## 3. Results and Discussion

### 3.1. Flour Properties

Total starch ranged from 40.53 ± 0.86 g/100 g dm (CF) to 46.63 ± 0.94 g/100 g dm (RCF) and 48.51 ± 1.0 g/100 g dm (DCF) for chickpea flours, while it was significantly (*p* < 0.05) higher (82.25 ± 1.48 g/100 g dm) in RF. On the other hand, as reported in our previous study [12], chickpea flours showed higher protein (21–23.5 g/100 g dm), fat (5.7–7.6 g/100 g dm), and ash (2.3–3.1 g/100 g dm) contents than RF (8.29 g/100 g dm, 1.3 g/100 g dm, and 0.7 g/100 g dm, respectively). Furthermore, according to the product datasheet, RF had a very low fiber content as it was obtained by polished rice, while CF, RCF, and DCF had a fiber content of 23, 17–20, and 10 g/100 g, respectively, confirming that roasting and dehulling processes partially remove chickpea hulls.

### 3.2. Technological Properties of Bread

Bake loss, height, specific volume, and color of fresh bread are reported in Table 1. RCF, CF, and DCF containing bread formulations had slightly higher bake loss values (*p* > 0.05). Regarding the specific volume, as attended, the RF+CF bread showed a lower volume than RF bread, presumably due to the presence of fibers (Section 3.1) that somehow interfered with the protein networking. Accordingly, bread containing RCF that was thermally treated and processed by partially removing hulls [12] exhibited significantly (*p* < 0.05) higher volumes compared to RF+CF bread; this can also be attributed to the significantly (*p* < 0.05) higher gas retention coefficient of RF+RCF dough (98.60 ± 0.5%) compared to RF+CF dough (97.70 ± 0.3%) as evaluated with a Chopin Rheofermentometer F3 (Chopin, Villeneuve-La-Garenne, Cedex, France) in our previous study [12]. This result is also in accordance with the findings of Ouazib et al. [22], where breads made with 100% toasted chickpea flours had higher specific loaf volume than breads prepared with raw, germinated, and cooked chickpea flours. Contrary, Burešová et al. [20] found that there was no significant difference between the specific volumes of rice and chickpea breads, while Aguilar et al. [19] observed only a slight increase in specific volume upon the addition of 7.8% chickpea flour to cornstarch-based GF bread formulation. Miñarro et al. [21] showed that chickpea flour added to cornstarch-based GF bread resulted in an enhanced specific volume that was attributed to the high foam stabilizing capacity of chickpea protein. These contradictory findings reported in literature are mainly due to the different amounts of chickpea flour added into the formulations and the type of flours in the blend.

The moisture content of fresh bread samples were 42.92 ± 0.96%, 42.97 ± 0.09%, 43.01 ± 0.48% and 40.15 ± 0.05% for RF, RF+RCF, RF+CF and RF+DCF, respectively (Table 1). Only RF+DCF had significantly lower moisture content than all the other samples (*p* < 0.05). At the end of the storage time of 95 h, low moisture loss values were obtained as the moisture content values were 41.90 ± 0.06%, 42.11 ± 0.76%, 41.62 ± 0.44%, and 38.57 ± 0.43% for RF, RF+RCF, RF+CF, and RF+DCF, respectively (Supplemental Table S1). Moreover, the water activity values of the fresh crumb samples were not significantly affected (*p* > 0.05) by flour types (0.994 ± 0.006, 0.989 ± 0.006, 0.992 ± 0.007 and 0.993 ± 0.007 for RF, RF+RCF, RF+CF, and RF+DCF, respectively). No significant differences were observed within each sample during storage and between the samples at 23, 47, 71, and 95 h of storage (*p* > 0.05) (Supplemental Table S1), suggesting the flours and HPMC used were able to bind water and the polyethylene bags used to store bread samples helped to prevent water loss.

As attended, according to the flours used in the formulation, different crumb lightness, redness, and yellowness indices were found (Table 1). In particular, RF+RCF showed L* values significantly (*p* < 0.05) lower than the other breads. This result was expected since RCF had the darkest color among flours used [35]. The dark color can be attributed to the roasting process to which the chickpea was subjected. As regards the a* values, RF+CF had a significantly (*p* < 0.05) lower a^*^ value than RF and RF+RCF. The index that discriminates most of the bread crumb color according to the flour used was b*. All formulations had significantly different b* values (*p* < 0.05): RF had the lowest one, RF+RCF had the highest, and RF+DCF had the lowest yellowness value among chickpea-containing breads. The crust lightness values were significantly different for RF+RCF, RF+CF, and RF+DCF when compared with RF (*p* < 0.05). Both the formulation and the process (baking condition) could affect the crust color. However, since the baking condition was standardized among the four samples, the differences evidenced among the four bread samples can be reasonably attributed to the flour used. In fact, due to the white color of rice flour, RF had the lightest crust. All the chickpea-containing breads showed an increase in crust darkness. Furthermore, the addition of chickpea flour, which is rich in protein, certainly contributed to the Maillard reactions that took place on the crust during baking. The crust redness was significantly different for all samples (*p* < 0.05): lowest for RF and highest for RF+DCF. The addition of chickpea flours increases the yellowness value regardless of the type of chickpea flours used (*p* < 0.05).

**Figure 1:**
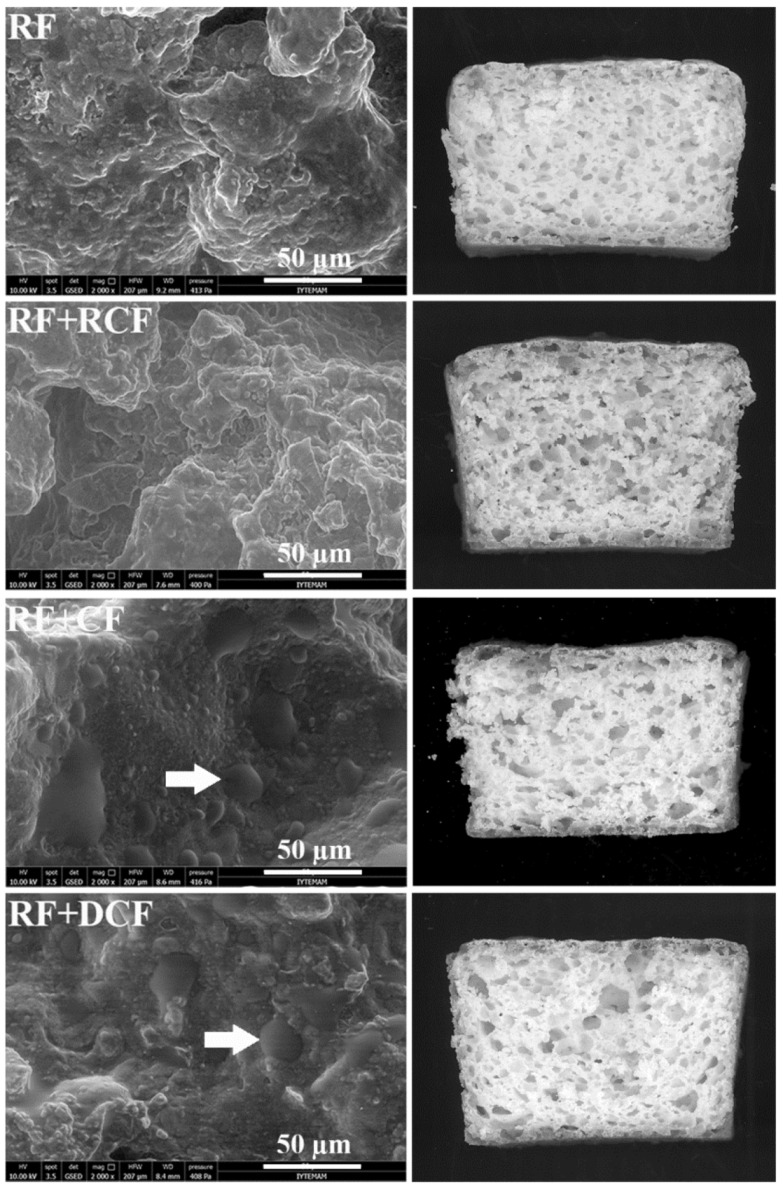
Crumb microstructure (Magnification ×2000) and cross-section of gluten-free bread (RF, rice flour; RCF, roasted chickpea flour; CF, raw chickpea flour; DCF, dehulled chickpea flour). The starch granules are shown with a white arrow.

Cross-sections of gluten-free bread slices and crumb microstructure are reported in Figure 1. RF+CF and RF+DCF bread samples exhibited high amounts of round-shaped intact starch granules (shown in Figure 1 with white arrow) with large sizes. This indicates that the starch granules were partially gelatinized during baking. The average starch granule sizes of rice flour (10 μm), raw (22 μm), roasted (19 μm), and dehulled (19 μm) chickpea flours was observed in our previous study [12]. Intact starch granules were also previously observed in buckwheat, quinoa, sorghum, teff, and wheat breads [36]. The partial gelatinization of starch granules was attributed to the limited and decreasing water levels during bread baking [37] and to the absence of thermal treatment of the flour since CF and DCF were only mechanically processed. On the contrary, RF+RCF bread showed more “fuse” and continuous structure where intact starch granules are less defined (suggesting a stronger gelatinization of the granules occurred); this is coherent with the subsequent tempering, moistening, resting, and roasting steps applied during leblebi production [12,13,14,15,16,17,18,19,20,21,22,23,24].

**Figure 2:**
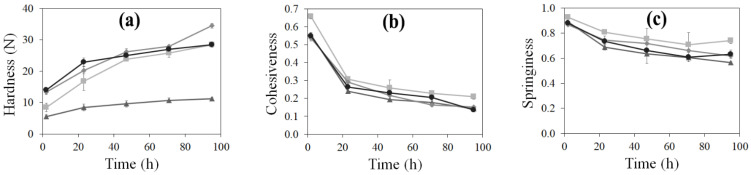
Evolution of crumb texture ((**a**) hardness (N); (**b**) cohesiveness; (**c**) springiness) during storage. RF (■), RF + CF (♦), RF+RCF (▲) and RF + DCF (●); RF, rice flour; CF, raw chickpea flour; RCF, roasted chickpea flour; DCF, dehulled chickpea flour.

The differences evidenced by ESEM were also reflected at the macroscopic level. In fact, different crumb porosity and pore distribution (Figure 1 and Table 2, respectively) were evidenced through image analysis. All breads exhibited high porosity values (above 40% vs. 22–26% of published GF bread data [25]); in particular, RF+RCF had the highest porosity and RF+CF and RF+DCF the lowest. High porosity values of RF+RCF possibly improved the specific volume (Table 1) and hardness (Figure 2) of this GF bread sample. Furthermore, according to pore size distribution, RF presented the highest amount of pores belonging to the 5–10 mm^2^ class, while chickpea flour addition led to the formation of a higher amount of small size pores. In fact, in chickpea flour containing breads, about 45% of pores had a dimension lower than 0.5 mm^2^; this can be attributed to the well-known foaming capacity of chickpea flours in respect to RF [12].

According to crumb microstructure and porosity, different texture properties are expected. Since quick staling of GF bread is one of the main problems still to be solved, bread crumb was subjected to a TPA after 2 hours from baking (fresh bread) and at different storage times up to 95 h in order to evaluate the staling process (Figure 2). RF+CF and RF+DCF fresh breads presented significantly (*p* < 0.05) higher hardness (13.37 ± 0.87 N and 14.05 ± 0.57 N, respectively) compared to RF (8.42 ± 1.38 N) and RF+RCF (5.49 ± 0.58 N) samples. In particular, bread containing RCF presented a softer crumb even if the moisture content of this bread (42.97 ± 0.09%) was not significantly different from that of RF (42.92 ± 0.96%) and RF+CF (43.01 ± 0.48%). This behavior could be related to the higher bread crumb porosity of this sample. Also, for the other samples, higher porosity corresponds to lower hardness values; RF+CF and RF+DCF had similar porosity and hardness values, although dehulled chickpea flour had lower fiber content. During the first 23 h of storage, the rate of increase in firmness was rapid for all samples. However, the hardening of RF+RCF was significantly lower than other breads. After 95 h, hardness values of the breads resulted as the corresponding values, i.e., RF+RCF, 11.22 ± 0.34 N; RF+DCF, 28.42 ± 0.08 N; RF, 28.45 ± 0.73 N; RF+CF, 34.53 ± 0.74 N. Thus, RF+RCF was 1.5 times softer than RF after 2 hours of baking and 2.5 times at the end of the storage period. According to Ouazib et al. [22], a relatively softer crumb was obtained for toasted (at 180 °C for 20 min on a stove) chickpea flour bread (167 N) compared to raw chickpea bread (251 N). In comparison with RF+RCF, which contained roasted chickpea flour, the toasted chickpea bread prepared in [22] exhibited 15 times higher hardness. The differences in the composition of the flour used (100% chickpea), and the intensity of the heat treatment applied to chickpea may account for the differences in the hardness values. In fact, in Ouazib et al. [22], heat was applied to chickpeas only once (180 °C, 20 min) during roasting. On the contrary, in our study, the roasted chickpea grains were obtained after at least three tempering and one roasting steps that were interspersed by resting and moistening processes as explained in Coşkuner and Karababa [24]. The rate of staling was the highest for RF (2.38) and lowest for RF+DCF (1.02) and RF+RCF (1.04), while RF+CF had a moderate staling rate (1.58). Bread staling is a complex phenomenon that involves mainly the migration of water from crumb to crust and starch retrogradation [38,39]. Apart from starch, other bread components have been thought to affect bread staling as they interact with water. For instance, the presence of protein (i.e., gluten) was found to contribute to the softness of the crumb by increasing the amount of water that plasticizes [40]. In some of the studies, the effect of gluten on staling was linked with the dilution effect on starch [41]. On the other hand, the importance of the interactions between starch–gluten and starch–starch rather than their presence or absence was suggested as the most important point [42,43]. Consequently, in our study, the lower staling rate of chickpea flour-containing GF breads vs. RF bread could be attributed to the higher (24–36%) protein content of these breads (Table 3). Moreover, the lower amount of starch and the higher fiber content of chickpea flours (in particular DCF and RCF) in comparison with RF (Section 3.1) may also positively affect the staling rate of the breads [44].

Cohesiveness is an indicator of the internal resistance of bread to a double compression. A high level of cohesiveness is desirable in order to obtain a less crumbly bread structure, a typical defect of gluten-free bread. In fact, during mastication, less cohesive bread starts to fractionate in the mouth [45]. As seen in Figure 2b, the fresh breads containing chickpea flours showed significant (*p* < 0.05) lower cohesiveness values (0.54–0.56) in comparison to rice bread (0.66 ± 0.01). Sharp decreases in the cohesiveness values of all crumbs were observed during storage, particularly in the first 23 h. During storage, cohesiveness values progressively decreased down to 0.21 ± 0.01 for RF, 0.14 ± 0.0.01 for RF+RCF, 0.15 ± 0.00 for RF+CF, and 0.14 ± 0.01 for RF+DCF after 95 h. In comparison to the cohesiveness of breads containing toasted chickpea flours [22], the cohesiveness values of our samples were higher. Similarly to our results, Burešová et al. [20] reported that rice bread exhibited a higher cohesiveness value (0.76) compared to chickpea bread (0.58), highlighting the negative effect of chickpea flour addition on cohesiveness. Moreover, decreasing cohesiveness values were also observed when high pea protein powder (almost 20%, flour-based) was added in the buckwheat–flaxseed based GF bread formulations [46]. Accordingly, the replacement of 25% of RF with the chickpea flours having higher protein content (Section 3.1) determined a decrease of the bread cohesiveness (Figure 2), presumably due to more rigidity of the starch–protein network.

Crumb springiness is considered an indicator of the elasticity of the bread crumb. When compared to RF, presence of chickpea flours (RCF, CF and DCF) in bread formulations caused a slight but statistically significant decrease in springiness from 0.93 ± 0.01 (RF) to 0.89 ± 0.01 (RF+RCF), 0.87 ± 0.01 (RF+CF), and 0.88 ± 0.02 (RF+DCF). Ouazib et al. [22] reported similar-to-lower springiness values for fresh bread containing raw (0.85) and toasted (0.70) chickpea flour. On the contrary, increased springiness values were observed in GF rice muffins containing protein isolate [47]. After 95 h of storage, springiness values of breads decreased to 0.74 ± 0.02 for RF, 0.56 ± 0.0.01 for RF+RCF, 0.62 ± 0.01 for RF+CF, and 0.63 ± 0.03 for RF+DCF.

### 3.3. Nutritional Properties of Bread

The composition of breads is shown in Table 3 together with the results of rapidly (RDS) and slowly (SDS) digestible starch fractions. The substitution of 25% of rice flour with differently processed chickpeas flours increased the protein content by 33.9% (CF), 35.9% (RCF), and 23.7% (DCF). Moreover, a significant (*p* < 0.05) increase in the amount of fat and phenolic compounds was noticed in chickpea breads when compared to rice flour bread.

The TPC of flour samples were 44.55 ± 4.94 mg GAE/100 g dm for rice flour, 119.63 ± 0.85, 118.32 ± 3.56, and 115.98 ± 4.52 mg GAE/100 g dm for roasted, raw, and dehulled chickpea flour, respectively. These results were close to the values in literature where TPC contents of commercial raw chickpea samples were reported as 141 ± 8 mg GAE/100 g [48] and 157 ± 6–147 ± 7 mg GAE/100 g dm [49]. In comparison to rice flour, chickpea flours had significantly higher (*p* < 0.05) TPC values regardless of the type of processing applied. However, no significant change (*p* > 0.05) was observed among the raw, roasted, and dehulled chickpea flours. Unlike our findings, Fares and Menga [50] reported a significant increase in TPC after toasting chickpea flour, which was attributed to the compounds such as Amadori and Heyns products formed during toasting. Toasting also increased the TPC of buckwheat grains [51]. The TPC values of RF+RCF, RF+CF, and RF+DCF breads were significantly different (*p* < 0.05) than RF (Table 3): RF had the lowest TPC (49.36 ± 2.47 mg GAE/100 g dm), followed by RF+CF (65.29 ± 2.25 mg GAE/100 g dm), RF+DCF (71.87 ± 2.05 mg GAE/100 g dm) and RF+RCF (80.52 ± 5.13 mg GAE/100 g dm). When the TPC contents of chickpea flour-containing breads were compared, the TPC content of RF+RCF bread was significantly (*p* < 0.05) higher than RF+CF bread.

The total available starch levels were significantly higher for RF (86.94 ± 0.60 g/100 g dm) when compared to chickpea flour-containing breads (*p* < 0.05). Among the chickpea containing breads, RF+CF (75.79 ± 0.90 g/100 g dm) and RF+RCF (75.20 ± 0.59 g/100 g dm) had significantly lower available starch contents than RF+DCF (78.43 ± 1.29 g/100 g dm) (*p* < 0.05). These values were in accordance with Gularte et al. [52].

RDS and SDS are indicative of high and low glycemic responses, and so as high and low glycemic index, respectively [53]. The Glycemic index (GI) term is used to evaluate the blood glucose response of foods. According to GI values, foods are classified as low (<55), medium (55–69), or high (>70) GI foods [54]. The nature of starch, monosaccharide components, food processing, heat treatment/cooking, and food components such as fat and protein play an important role in the GI [55]. In our study, significant differences in digestible starch fractions were observed between bread samples. In particular, all the differently treated chickpea containing bread samples (RF+CF, RF+RCF, and RF+DCF) had significantly (*p* < 0.05) lower RDS fractions than those determined in RF. The possible explanation for this might be the presence of dietary fiber in chickpea flours and the higher amylose content of chickpea starches (30–35% on average) [56,57,58] compared to rice flour (26.2 g/100 g dm) [44]. Interestingly, when compared to RF, only RF+DCF had a significantly (*p* < 0.05) higher SDS fraction; this feature may be of interest from the nutritional point of view since it could reduce the glycemic potential [59] of the bread. Wolter et al. [60] reported a key role of factors such as the size of starch granules, degree of gelatinization, starch composition, protein, and lipid content/interactions in affecting the starch digestibility of bread. The relatively high SDS content in DCF containing bread might be related to the presence of intact starch granules in breads (Figure 1). Indeed, the glycemic response appears to be directly related to the amount of RDS, and the insulin demand is inversely correlated to the SDS fraction [61]. In general, RDS represents the prevailing fraction in GF products [52,62].

**Figure 3:**
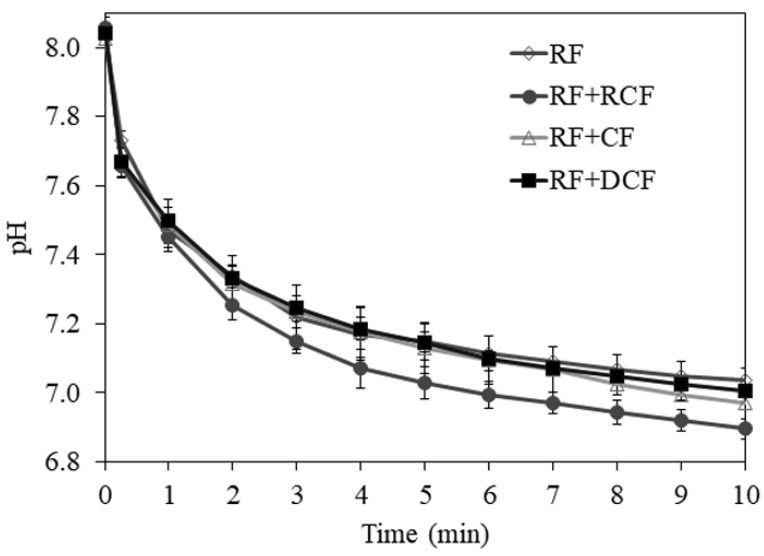
pH drop during in vitro protein digestion of breads (RF, rice flour; RCF, roasted chickpea flour; CF, raw chickpea flour; DCF, dehulled chickpea flour).

Figure 3 reports pH drop caused by the increase in H+ ions due to the breakdown of peptide bonds and formation of free carboxyl groups of amino acids [63] during in vitro protein digestion of breads. An improved digestibility for roasted chickpea bread (85.57 ± 0.52%; *p* < 0.05) in comparison with RF (83.07 ± 0.64%) was observed, while only small changes resulted for RF+CF (84.25 ± 0.04%), and RF+DCF (83.58 ± 0.29%) (Table 3). It was observed that bread prepared with cooked chickpea flour had significantly (*p* < 0.05) higher in vitro protein digestibility values compared to raw, germinated, and toasted chickpea breads [22]; similar to our findings, no significant differences (*p* > 0.05) was observed among breads made with raw, germinated, and toasted chickpea flours. The increased digestibility of RF+RCF has been attributed to the protein denaturation that occurred during the cooking process [22]. Protein digestibility is one of the useful measurements for evaluating protein quality and availability. Low protein digestibility has been linked to anti-nutritional factors, such as protease inhibitors, particularly present in legumes [64], and the presence of tannins and phytic acids in the seed. The effect of heat treatment on the reduction of enzyme inhibitors and lectins is well-recognized in the literature [65,66]. In a review on protein digestibility of sorghum, processes such as dry heating, grain refinement, and fermentation were suggested to increase protein digestibility [67]. On the other hand, as the protein contents of the bread samples increased, in vitro protein digestibility values were also increased (Table 3). In agreement with this observation, it was found that [68] egg white protein and soy protein isolate addition improved the protein digestibility of gluten-free pasta based on banana flour.

## 4. Conclusions

The effects of the addition of raw, roasted, and dehulled chickpea flour on the technological and nutritional characteristics of rice-based gluten-free bread were evaluated. Roasted chickpea flour addition resulted in breads with increased specific volume and darkness of crumb and crust. Among all the chickpea-containing breads, the one with added roasted chickpea flour showed a softer crumb and low staling rate during 95 h storage. Furthermore, a significant increase in protein, ash, and fat contents of the breads and reduced total available starch levels were achieved with the addition of chickpea flours to rice-based bread. Additionally, the total phenolic content of the breads significantly increased after chickpea flour addition, in particular in the bread containing roasted chickpea flour. In raw, roasted, and dehulled chickpea flour-containing breads, RDS levels were significantly (*p* < 0.05) lower than rice-based gluten-free bread and, consequently, SDS levels tended to increase, suggesting a potential effect in reducing the potential glycemic response of these breads. The protein digestibility was enhanced in roasted chickpea flour-containing bread.

In the light of this study, it was revealed that rice breads enriched with 25% chickpea flours displayed varying and, in general, improved technological and nutritional properties. Furthermore, the results of the study evidenced that a by-product of leblebi snack manufacturing (i.e., RCF) can be successfully valorized in GF bread formulations as a cheap, sustainable, and nutritious ingredient.

## Figures and Tables

**Table 1:** Technological properties of fresh gluten-free breads.

Sample	Bake Loss (%)	Height Max (cm)	Specific Volume (cm^3^/g)	Moisture (g/100 g)	Crumb Color			Crust Color		
					L*	a*	b*	L*	a*	b*
RF	19.62 ± 0.47 ^a^	4.69 ± 0.23 ^ab^	2.63 ± 0.11 ^ab^	42.92 ± 0.96 ^a^	75.07 ± 0.05 ^a^	-2.21 ± 0.19 ^a^	7.75 ± 0.07 ^d^	72.48 ± 1.08 ^a^	-0.50 ± 0.17 ^d^	24.55 ± 0.35 ^b^
RF + CF	20.44 ± 0.18 ^a^	4.34 ± 0.03 ^b^	2.51 ± 0.11 ^b^	43.01 ± 0.48 ^a^	75.47 ± 0.39 ^a^	-3.13 ± 0.21 ^b^	16.47 ± 0.22 ^b^	52.90 ± 0.39 ^b^	8.58 ± 0.08 ^b^	32.86 ± 0.53 ^a^
RF + RCF	20.43 ± 0.74 ^a^	5.00 ± 0.07 ^a^	2.89 ± 0.03 ^a^	42.97 ± 0.09 ^a^	70.56 ± 1.16 ^b^	-2.02 ± 0.04 ^a^	20.59 ± 1.00 ^a^	56.02 ± 0.53 ^b^	7.14 ± 0.20 ^c^	33.49 ± 0.17 ^a^
RF + DCF	20.77 ± 0.57 ^a^	4.66 ± 0.04 ^ab^	2.75 ± 0.04 ^ab^	40.15 ± 0.05 ^b^	75.09 ± 0.02 ^a^	-2.71 ± 0.22 ^ab^	13.42 ± 0.09 ^c^	48.96 ± 1.14 ^c^	9.80 ± 0.03 ^a^	31.30 ± 1.08 ^a^

RF, rice flour; CF, raw chickpea flour; RCF, roasted chickpea flour; DCF, dehulled chickpea flour. Mean values having different letters in the same column are significantly different (*p* < 0.05). Values are mean ± SD.

**Table 2:** Crumb porosity (%) and cell size distribution (%) of gluten-free bread crumbs.

Sample	Crumb Porosity (%)	Cell Dimension					
		0.1–0.2 (mm^2^)	0.2–0.5 (mm^2^)	0.5–1 (mm^2^)	1–5 (mm^2^)	5–10 (mm^2^)	>10 (mm^2^)
RF	45.36 ± 2.87 ^ab^	18.03 ± 0.44 ^b^	18.84 ± 3.90 ^a^	15.37 ± 1.52 ^a^	33.80 ± 2.63 ^a^	8.21 ± 1.34 ^a^	5.44 ± 1.46 ^a^
RF + CF	41.49 ± 0.11 ^b^	23.02 ± 0.21 ^a^	21.69 ± 0.91 ^a^	17.17 ± 1.17 ^a^	27.92 ± 0.67 ^b^	4.76 ± 0.05 ^b^	5.43 ± 1.15 ^a^
RF + RCF	51.41 ± 2.30 ^a^	23.16 ± 0.07 ^a^	22.23 ± 1.42 ^a^	16.70 ± 0.61 ^a^	25.81 ± 0.11 ^b^	5.33 ± 0.20 ^b^	6.77 ± 1.66 ^a^
RF + DCF	41.84 ± 1.25 ^b^	21.99 ± 0.41 ^a^	23.04 ± 1.04 ^a^	17.21 ± 0.42 ^a^	29.07 ± 0.15 ^ab^	5.29 ± 0.01 ^b^	3.40 ± 0.34 ^a^

RF, rice flour; CF, raw chickpea flour; RCF, roasted chickpea flour; DCF, dehulled chickpea flour. Mean values having different letters in the same column are significantly different (*p* < 0.05). Values are mean ± SD.

**Table 3:** Nutritional properties of gluten-free breads.

Bread	Protein (g/100 g dm)	Fat (g/100 g dm)	Ash (g/100 g dm)	TPC (mg GAE/100 g dm)	RDS (g/100 g dm)	SDS (g/100 g dm)	Total Available Starch (g/100 g dm)	%RDS (g/100 g Available Starch)	%SDS (g/100 g Available Starch)	IVPD (%)
RF	9.72 ± 0.08 ^b^	1.61 ± 0.38 ^c^	2.01 ± 0.03 ^b^	49.36 ± 2.47 ^c^	81.04 ± 0.31 ^a^	5.90 ± 0.62 ^bc^	86.94 ± 0.60 ^a^	93.2 ± 0.7 ^a^	6.8 ± 0.7 ^c^	83.07 ± 0.64 ^b^
RF + CF	13.02 ± 0.14 ^a^	4.78 ± 0.10 ^b^	2.78 ± 0.09 ^a^	65.29 ± 2.25 ^b^	68.81 ± 0.96 ^b^	6.98 ± 0.49 ^b^	75.79 ± 0.90 ^c^	90.8 ± 0.7 ^b^	9.2 ± 0.7 ^b^	84.25 ± 0.04 ^ab^
RF + RCF	13.21 ± 0.03 ^a^	4.58 ± 0.07 ^b^	2.45 ± 0.01 ^ab^	80.52 ± 5.13 ^a^	69.87 ± 0.61 ^b^	5.33 ± 0.60 ^c^	75.20 ± 0.59 ^c^	92.9 ± 0.8 ^ab^	7.1 ± 0.8 ^c^	85.57 ± 0.52 ^a^
RF + DCF	12.03 ± 0.71 ^a^	5.86 ± 0.16 ^a^	2.63 ± 0.21 ^a^	71.87 ± 2.05 ^ab^	67.29 ± 1.63 ^b^	11.14 ± 0.43 ^a^	78.43 ± 1.29 ^b^	85.8 ± 0.7 ^c^	14.2 ± 0.7 ^a^	83.58 ± 0.29 ^b^

RF, rice flour; CF, raw chickpea flour; RCF, roasted chickpea flour; DCF, dehulled chickpea flour; TPC, total phenolic content; RDS, rapidly digestible starch; SDS, slowly digestible starch; total available starch (RDS+SDS); IVPD, in vitro protein digestibility; dm, dry matter. Means having different letters at the same column are significantly different (*p* < 0.05). Values are mean ± SD.

## Data Availability

The datasets generated for this study are available on request to the corresponding author.

## Supplementary Materials

The following are available online at https://www.mdpi.com/article/10.3390/foods11020199/s1, Figure S1: Representative force (g) versus time (s) curve of TPA; Table S1: Changes in moisture content (g/100g) and water activity of breads during 95 hours of storage.
