# Supplementary material for: Impact of Raw, Roasted and Dehulled Chickpea Flours on Technological and Nutritional Characteristics of Gluten-Free Bread
Source: Foods. 2022 Jan 12;11(2):199. doi: 10.3390/foods11020199 (PMC8774402; doi:10.3390/foods11020199)
Supplement: Supplementary file 1 [file foods-11-00199-s001.zip › foods-1463141-supplementary.pdf]

Foods **2022**, *11*, 199. https://doi.org/10.3390/foods11020199

Impact of Raw, Roasted and Dehulled Chickpea Flours on Technological and Nutritional Characteristics of Gluten-Free Bread

Gokcen Kahraman, Sebnem Harsa, Maria Cristina Casiraghi, Mara Lucisano and Carola Cappa

Table S1. Changes in moisture content (g/100g) and water activity of breads during 95 hours of storage.

	Time (h)	RF	RF+CF	RF+RCF	RF+DCF
Moisture (g/100g)	2	42.92 ± 0.96^{Aa}	43.01 ± 0.48^{Aa}	42.97 ± 0.09^{Aa}	40.15 ± 0.05^{Ab}
	23	42.74 ± 0.93^{Aa}	42.75 ± 0.30^{ABa}	42.80 ± 0.17^{Aa}	39.94 ± 0.16^{Ab}
	47	42.11 ± 0.73^{Aa}	41.95 ± 0.05^{ABCa}	42.38 ± 0.19^{Aa}	39.41 ± 0.34^{Ab}
	71	41.30 ± 0.40^{Aa}	41.21 ± 0.12^{Ca}	41.63 ± 0.01^{Aa}	38.65 ± 0.71^{Ab}
	95	41.90 ± 0.06^{Aa}	41.62 ± 0.44^{BCa}	42.11 ± 0.76^{Aa}	38.57 ± 0.43^{Ab}
Moisture loss during storage (g/100g)		2.39	3.22	2.00	3.94
Water Activity	2	0.994 ± 0.006	0.992 ± 0.007	0.989 ± 0.006	0.993 ± 0.007
	23	0.995 ± 0.002	0.992 ± 0.007	0.988 ± 0.012	0.991 ± 0.007
	47	0.993 ± 0.007	0.991 ± 0.010	0.987 ± 0.011	0.992 ± 0.007
	71	0.996 ± 0.005	0.991 ± 0.007	0.989 ± 0.001	0.991 ± 0.004
	95	0.993 ± 0.001	0.988 ± 0.003	0.987 ± 0.006	0.987 ± 0.004

RF, rice flour; CF, raw chickpea flour; RCF, roasted chickpea flour; DCF, dehulled chickpea flour. $^{A-D}$ Means having different letters in the same column are significantly different ($P < 0.05$). $^{a-c}$ Means having different letters in the same row are significantly different ($P < 0.05$). No significant differences ($P > 0.05$) observed for water activity values. Values are mean ± SD.

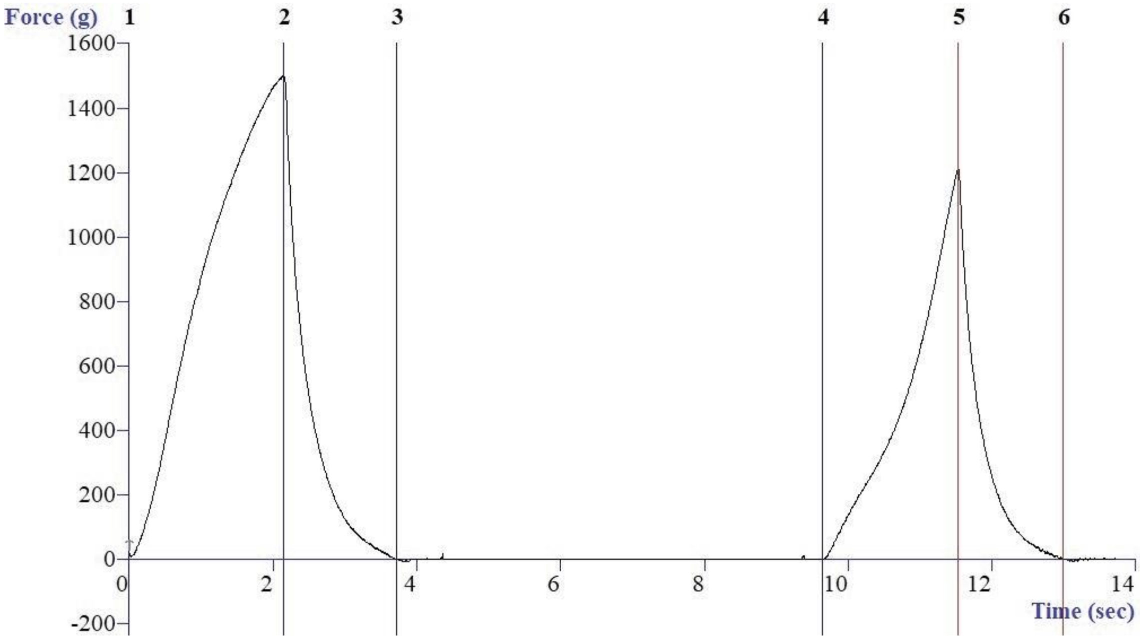

Figure S1. Representative force (g) versus time (s) curve of TPA
